# Development and validation of a multimodal nomogram integrating maternal serum angiogenic markers and fetal hemodynamics for risk stratification of late-onset fetal growth restriction

**DOI:** 10.3389/fmed.2026.1863129

**Published:** 2026-10-29

**Authors:** Yaqin Li, Jie Huang, Xuan Zhang, Xueyan Shen, Botao Yang, Liman Fu, Wei Zhang, Wei Hao, Fang Li

**Affiliations:** 1 Fourth Hospital of Shijiazhuang, Shijiazhuang, China; 2 Health Commission of Hebei Province, Shi Jiazhuang, China

**Keywords:** cerebroplacental ratio, clinical utility, late-onset fetal growth restriction, machine learning, nomogram, sFlt-1/PlGF ratio

## Abstract

**Background:**

Late-onset fetal growth restriction (L-FGR) remains a major challenge in obstetrics due to its subtle clinical presentation and high risk of adverse perinatal outcomes. This study aimed to develop and validate a multimodal nomogram integrating maternal serum biomarkers and fetal ultrasound parameters to improve the diagnostic evaluation and risk stratification of L-FGR and subsequent adverse endpoints.

**Methods:**

This retrospective nested case-control study included 300 singleton pregnancies (100 L-FGR cases and 200 matched normal controls) managed at our institution. Fetal hemodynamic indices, biometry, and maternal serum levels of PlGF and the sFlt-1/PlGF ratio were retrospectively analyzed. Independent predictors were identified via multivariate logistic regression and confirmed by LASSO regression. A multimodal diagnostic-support and risk-stratification nomogram was constructed and evaluated for discrimination, calibration, and clinical utility.

**Results:**

The sFlt-1/PlGF ratio, cerebroplacental ratio (CPR), abdominal circumference (AC) percentile, and PlGF were identified as independent predictors. The combined multimodal model demonstrated superior discrimination, achieving an AUC of 0.948 (95% CI: 0.918–0.978) in the training cohort (*n* = 210) and maintaining robust discrimination with an AUC of 0.910 (95% CI: 0.845–0.965) in the independent internal validation cohort (*n* = 90), significantly outperforming single-modality models (*P* < 0.05). Calibration curves and the Hosmer-Lemeshow test (*P* = 0.601) indicated robust fit. Decision curve analysis (DCA) and Net Reclassification Index (NRI = 0.452) confirmed substantial clinical net benefit. Furthermore, the high-risk group stratified by the nomogram exhibited significantly accelerated time-to-delivery (Log-rank *P* < 0.001) and remarkably higher incidences of severe composite adverse perinatal outcomes, specifically NICU admission, emergency cesarean section, and neonatal hypoglycemia. Benchmarking revealed that our interpretable logistic regression model achieved comparable efficacy to complex ML algorithms like XGBoost.

**Conclusion:**

We successfully developed a highly robust, multimodal nomogram that synergistically combines hemodynamic and biochemical markers. This tool facilitates accurate, individualized L-FGR identification and risk stratification, effectively guiding clinical decision-making to mitigate adverse perinatal outcomes.

## 1 Introduction

Late-onset fetal growth restriction (L-FGR), generally defined as a failure of the fetus to reach its intrinsic growth potential diagnosed after 32 weeks of gestation, remains one of the most insidious challenges in modern obstetrics (1, 2). Unlike early-onset FGR, which is strongly associated with severe placental insufficiency and easily detectable abnormal umbilical artery (UA) Doppler velocimetry, L-FGR often presents with subtle clinical manifestations (3). Despite its milder placental pathology, L-FGR accounts for the majority of FGR cases and contributes disproportionately to unexplained late stillbirths, neonatal neurodevelopmental morbidities, and adverse perinatal outcomes (4, 5).

Currently, the prenatal diagnosis of L-FGR relies heavily on two-dimensional ultrasound biometry, primarily the estimated fetal weight (EFW) and abdominal circumference (AC) (6). However, these traditional morphometric parameters lack sufficient sensitivity, as the fetal size in L-FGR may only marginally deviate from the population norm (7). To enhance diagnostic accuracy, fetal hemodynamic assessment, particularly the cerebroplacental ratio (CPR), has been introduced to detect the “brain-sparing effect”—a hallmark of fetal hypoxia (8, 9). While a reduced CPR correlates with adverse perinatal outcomes, its standalone predictive value is often compromised by high false-positive rates, leaving clinicians with a dilemma regarding the optimal timing of delivery to balance the risks of intrauterine demise and iatrogenic prematurity (10, 11).

Fundamentally, L-FGR is driven by placental malperfusion and an imbalance in angiogenic factors (12). Placental growth factor (PlGF), a pro-angiogenic protein, and soluble fms-like tyrosine kinase-1 (sFlt-1), an anti-angiogenic factor, are well-established biomarkers for preeclampsia (13, 14). Emerging evidence suggests that an elevated maternal serum sFlt-1/PlGF ratio also reflects the placental syncytiotrophoblast stress inherent in isolated FGR (15–17). Although utilizing the sFlt-1/PlGF ratio as an adjunctive diagnostic tool is gaining traction, comprehensive models that synergistically integrate these subclinical biochemical alterations with sonographic hemodynamic changes remain scarce (18, 19). Most existing predictive models are limited by a single modality, inadequate clinical utility validation, or a lack of user-friendly tools for daily triage (20).

Therefore, this retrospective nested case-control study aimed to develop and internally validate a novel, multimodal diagnostic-support nomogram integrating maternal serum biomarkers (PlGF, sFlt-1/PlGF ratio) and advanced fetal ultrasound parameters (e.g., AC percentile and CPR) to improve the diagnostic evaluation and risk stratification of L-FGR. Furthermore, we sought to extensively evaluate the clinical utility of this nomogram in predicting composite adverse perinatal outcomes, and to benchmark its methodological robustness against state-of-the-art machine learning algorithms.

## 2 Materials and methods

### 2.1 Study design and participants

This study employed a nested case-control design derived from an initial screening of 1,420 singleton deliveries managed at our institution between January 2023 and December 2025. Patients were consecutively evaluated, and individuals with multiple gestations (*n* = 42), known chromosomal or severe structural anomalies (*n* = 28), pre-existing chronic maternal diseases (*n* = 65), active TORCH or systemic infections (*n* = 15), and missing core Doppler or biomarker data (*n* = 50) were excluded. Among the remaining eligible cohort, 100 pregnant individuals strictly fulfilling the ISUOG diagnostic criteria for L-FGR were identified as the case group. Concurrently, 200 gestational-age-matched normal singleton pregnancies without hypertensive disorders or fetal growth abnormalities were randomly sampled at a 1:2 ratio to constitute the control group (Figure 1). The combined cohort (*N* = 300) was randomly partitioned at a 7:3 ratio into a training set (*n* = 210) and an internal validation set (*n* = 90). Because of this enriched 1:2 case-control sampling structure, absolute risk estimates, predictive values (PPV and NPV), and Decision Curve Analysis net benefits should be interpreted with appropriate caution when generalizing to unselected general obstetric populations with a lower natural prevalence. The study protocol was approved by the Institutional Review Board and Ethics Committee of Shijiazhuang Obstetrics and Gynecology Hospital (Approval No. 20230003). Due to the retrospective nature of the study and the use of anonymized clinical records, the requirement for written informed consent was formally waived.

**Figure 1:**
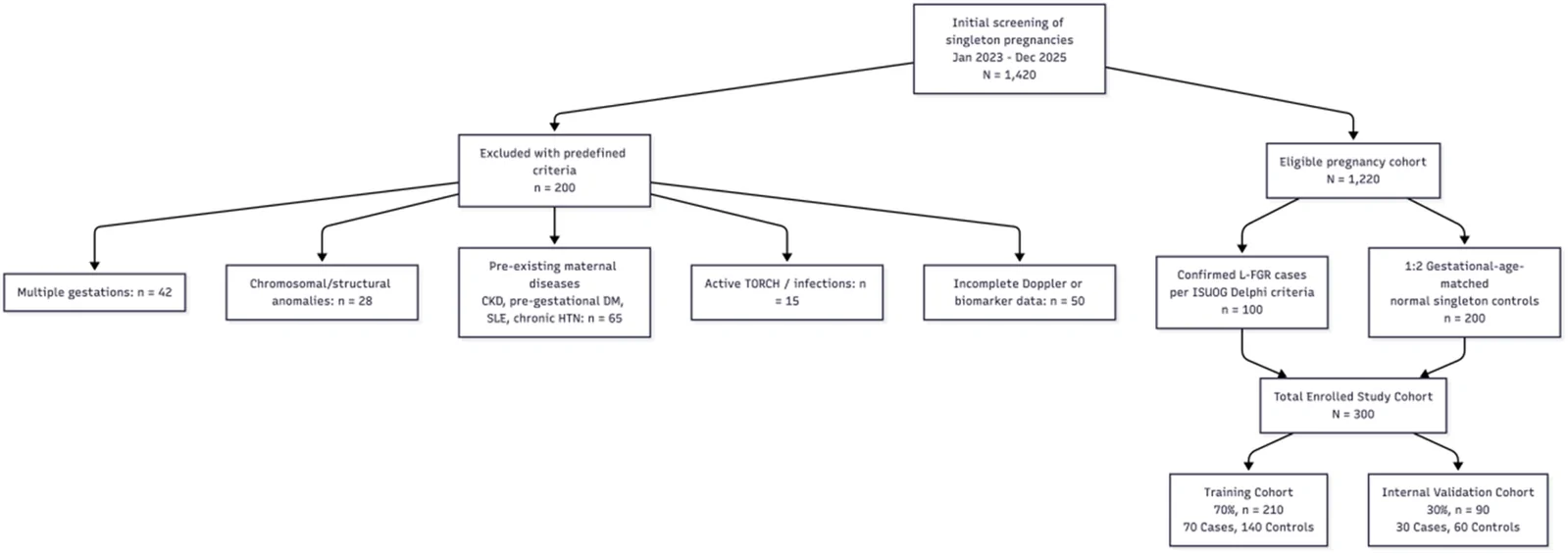
Flowchart of patient screening, enrollment, and cohort partitioning singleton deliveries were screened, 200 excluded, and 300 included (210 training, 90 validation)

### 2.2 Clinical data collection

Maternal baseline demographic characteristics, including maternal age, pre-pregnancy body mass index (BMI), parity, and detailed medical histories, were retrieved from the electronic medical record (EMR) system. Gestational age was determined based on the last menstrual period and corroborated by the first-trimester crown-rump length (CRL) measurement.

### 2.3 Fetal ultrasound and Doppler measurements

All obstetric ultrasound and Doppler examinations were performed by senior sonographers using a high-resolution ultrasound system (e.g., Voluson E8/E10, GE Healthcare, Zipf, Austria) equipped with a 2–8 MHz convex abdominal transducer. Biometric parameters, including biparietal diameter (BPD), head circumference (HC), abdominal circumference (AC), and femur length (FL), were measured to calculate the estimated fetal weight (EFW) using the Hadlock formula. The AC and EFW percentiles were determined according to local/international fetal growth standards.

For hemodynamic assessment, Doppler velocimetry was recorded during periods of fetal apnea and absence of fetal movement. The pulsatility index (PI) of the umbilical artery (UA) was measured in a free-floating loop of the umbilical cord. The middle cerebral artery (MCA) PI was measured at the proximal third of the MCA, near its origin from the circle of Willis. The cerebroplacental ratio (CPR) was calculated by dividing the MCA-PI by the UA-PI.

### 2.4 Retrieval of maternal serum biomarker data

Maternal peripheral venous blood samples were collected concurrently with third-trimester obstetric ultrasound examinations (gestational age≥32 weeks) at the time of suspected late-onset fetal growth restriction or routine late-pregnancy evaluation, and were entirely distinct from early-pregnancy aneuploidy screening protocols. For this retrospective study, maternal biomarker data were directly retrieved from the hospital's Laboratory Information System (LIS). The serum concentrations of Placental Growth Factor (PlGF) and soluble fms-like tyrosine kinase-1 (sFlt-1) had been quantitatively measured using an automated electrochemiluminescence immunoassay (ECLIA) on a cobas e-analyzer platform (Roche Diagnostics, Mannheim, Germany), in strict accordance with the manufacturer's standard operating procedures. The sFlt-1/PlGF ratio was subsequently calculated and recorded for each participant.

### 2.5 Definition of outcomes

The primary outcome was the diagnosis of Late-onset Fetal Growth Restriction (L-FGR). According to the Delphi consensus criteria established by the International Society of Ultrasound in Obstetrics and Gynecology (ISUOG), L-FGR was defined as a gestational age ≥32 weeks fulfilling at least one of the following: (1) EFW or AC < 3rd percentile; or (2) at least two of the following: EFW or AC < 10th percentile, EFW or AC crossing percentiles by > 2 quartiles, CPR < 5th percentile, or UA-PI > 95th percentile.

Secondary outcomes comprised a composite of adverse perinatal endpoints, defined as the occurrence of any of the following: (1) neonatal intensive care unit (NICU) admission; (2) emergency cesarean section due to non-reassuring fetal status/fetal distress; (3) confirmed neonatal hypoglycemia within 24 h postpartum.

### 2.6 Statistical analysis and machine learning benchmarking

All statistical analyses, feature selection, and model evaluations were performed using R software (version 4.2.1) and Python (version 3.9). Continuous variables were assessed for normality using the Shapiro–Wilk test. Normally distributed data were presented as mean ± standard deviation (SD) and compared using the independent Student's *t*-test. Non-normally distributed data were expressed as median [interquartile range (IQR)] and compared using the Mann–Whitney U test. Categorical variables were presented as frequencies (percentages) and evaluated using the Pearson's Chi-square test or Fisher's exact test.

To select optimal predictive features, variables with *P* < 0.05 in the univariate logistic regression were subjected to a multivariate logistic regression with backward stepwise selection. Additionally, Least Absolute Shrinkage and Selection Operator (LASSO) regression with 10-fold cross-validation was employed to prevent overfitting and confirm the robust feature subset. To account for physiological gestational fluctuations, gestational age at diagnosis/sampling was forced as a primary covariate in all multivariable logistic regression models. Continuous predictors were standardized prior to modeling: maternal serum PlGF and the sFlt-1/PlGF ratio were scaled and reported per 10-unit increase, whereas fetal Doppler and biometric indices were evaluated per original clinical unit. Multicollinearity among candidate predictors—particularly between PlGF and the sFlt-1/PlGF ratio—was formally examined using Variance Inflation Factors (VIF). All final predictors exhibited VIF values < 3.2, confirming the absence of severe multicollinearity. An individualized, visual nomogram was constructed based on the final multivariate logistic model.

The predictive performance was comprehensively evaluated using discrimination, calibration, and clinical utility metrics. Discrimination was quantified by the Area Under the Receiver Operating Characteristic curve (AUC) and Precision-Recall (PR) curves, with DeLong's test used for comparative AUC analysis. Calibration was assessed via calibration plots (1,000 bootstrap resamples) and the Hosmer-Lemeshow test. Clinical utility was demonstrated using Decision Curve Analysis (DCA), Clinical Impact Curves (CIC), and the Net Reclassification Index (NRI).

To evaluate the risk of adverse perinatal outcomes, patients were stratified into high- and low-risk groups based on the Youden index cutoff. Time-to-delivery was analyzed via Kaplan–Meier curves and compared using the Log-rank test. Finally, to validate the methodological ceiling, the performance of our logistic regression model was benchmarked against leading machine learning algorithms (XGBoost, Random Forest, and SVM). To guarantee a rigorous and unbiased comparison, hyperparameter optimization for all algorithms was executed within the training set using 5-fold cross-validation coupled with exhaustive grid search (tuning key parameters such as tree depth, learning rate, and regularization terms), rather than relying on default settings. A two-sided *P* < 0.05 was considered statistically significant.

## 3 Results

### 3.1 Baseline characteristics of the study population

A total of 300 singleton pregnancies were ultimately included in this study, comprising 100 patients diagnosed with late-onset fetal growth restriction (L-FGR group) and 200 normal pregnancies (Control group). The baseline clinical demographics, fetal ultrasound parameters, and maternal serum biomarkers are detailed in Table 1.

**Table 1:** Baseline clinical demographics, fetal ultrasound parameters, and serum biomarkers.

Variables	Control Group (*N* = 200)	L-FGR Group (*N* = 100)	*P*-value
Maternal Demographics			
Maternal Age (years, Mean ± SD)	31.4 ± 3.9	31.8 ± 4.2	0.435
Pre-pregnancy BMI (kg/m^2^, Mean ± SD)	24.1 ± 3.5	24.5 ± 3.7	0.380
Nulliparous, *n* (%)	110 (55.0%)	58 (58.0%)	0.812
Gestational age at diagnosis (weeks, Mean ± SD)	36.1 ± 1.3	34.2 ± 1.5	<0.001
Ultrasound Fetal Parameters			
AC percentile (%, Median [IQR])	55.0 [35.0–78.0]	4.5 [2.1–9.8]	<0.001
Cerebroplacental Ratio (CPR) (Median [IQR])	2.1 [1.7–2.6]	1.1 [0.8–1.4]	<0.001
UA-PI percentile (%, Mean ± SD)	48.0 ± 15.0	72.0 ± 18.0	<0.001
Maternal Serum Biomarkers			
PlGF (pg/mL, Median [IQR])	320.5 [180.2–480.0]	155.8 [90.5–230.1]	<0.001
sFlt-1/PlGF ratio (Median [IQR])	10.9 [5.4–19.2]	54.5 [28.0–91.3]	<0.001

There were no statistically significant differences between the two groups regarding maternal age, pre-pregnancy BMI, and parity distribution (all *P* > 0.05), indicating a well-balanced baseline. However, compared to the control group, fetuses in the L-FGR group exhibited significantly lower EFW and AC percentiles (*P* < 0.001). Hemodynamically, the CPR and Middle Cerebral Artery (MCA)-PI were significantly depressed, whereas the UA-PI was elevated in the L-FGR group (Figure 2C). Furthermore, maternal serum analysis revealed a dramatic reduction in PlGF levels and an abnormal elevation of the sFlt-1/PlGF ratio in the L-FGR cohort (*P* < 0.001) (Figures 2A,B).

**Figure 2:**
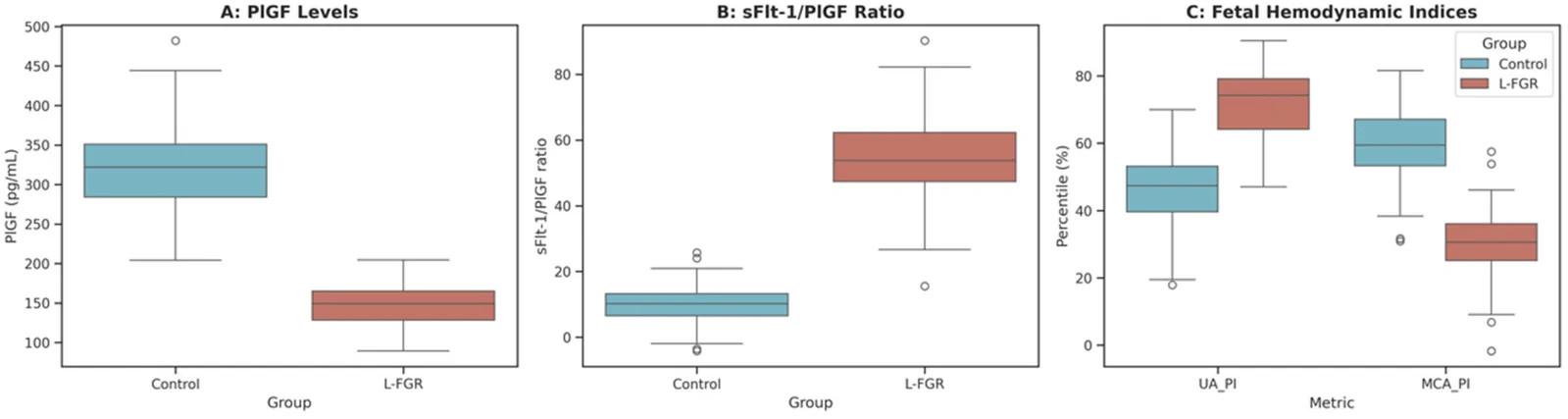
Baseline distribution of clinical, ultrasound, and serum biomarkers. **(A)** Box plots comparing maternal serum PlGF levels between the healthy control and L-FGR groups. **(B)** Comparison of the sFlt-1/PlGF ratio. **(C)** Comparison of fetal hemodynamic indices (UA-PI and MCA-PI percentiles).

### 3.2 Feature selection and independent associated factors

To identify robust predictors for L-FGR, we performed both univariate and multivariate logistic regression analyses (Table 2; Figure 3A). The univariate analysis indicated that maternal age, AC percentile, CPR, PlGF, and the sFlt-1/PlGF ratio were potential predictors. After adjusting for confounding variables in the multivariate model, the sFlt-1/PlGF ratio (Adjusted Odds Ratio [AOR] = 1.35, 95% CI: 1.15–1.58, *P* < 0.001) emerged as a significant independent risk factor. Conversely, higher PlGF, CPR, and AC percentiles were independently associated with reduced risk of L-FGR. Variable importance ranking further confirmed that serum biomarkers contributed the most significant predictive weight (Figure 3B).

**Figure 3:**
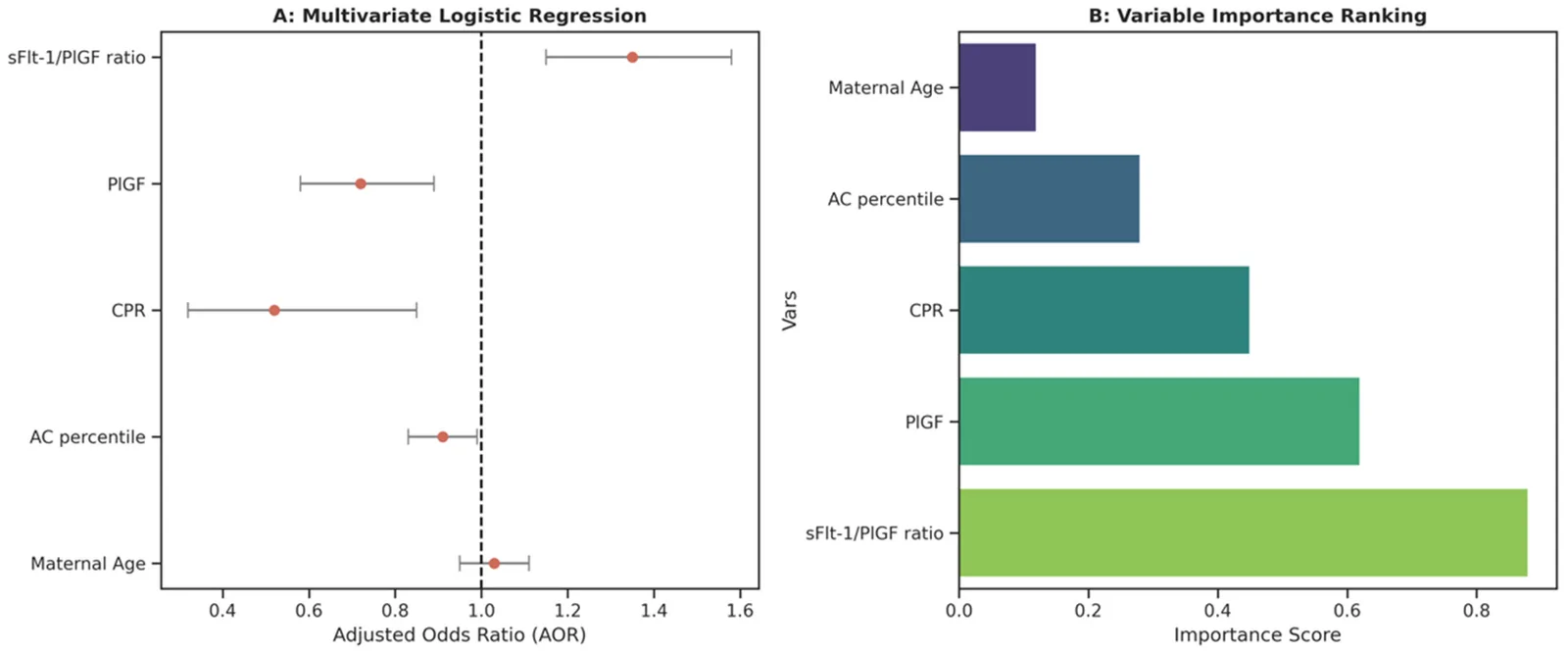
Feature selection and independent risk factor identification. **(A)** Forest plot of multivariate logistic regression analysis, identifying the sFlt-1/PlGF ratio, CPR, AC percentile, and PlGF as independent multivariable factors associated with L-FGR. **(B)** Variable importance ranking derived from the multivariable model, highlighting the substantial discriminative weight of maternal serum biomarkers.

**Table 2:** Univariate and multivariable logistic regression analysis for identifying factors associated with L-FGR in the training cohort (*n* = 210).

Predictor	Univariate OR (95% CI)	*P*-value	Multivariable AOR (95% CI)*	*P*-value	VIF
Gestational age at diagnosis (per week)	0.38 (0.30–0.49)	<0.001	0.44 (0.33–0.59)	<0.001	1.42
Maternal Age (per year)	1.05 (0.98–1.12)	0.175	1.02 (0.94–1.10)	0.584	1.08
AC percentile (per 1% increase)	0.88 (0.81–0.95)	<0.001	0.91 (0.83–0.99)	0.038	2.15
CPR (per 0.1-unit increase)	0.45 (0.28–0.72)	<0.001	0.54 (0.33–0.88)	0.012	2.38
PlGF (per 10 pg/mL increase)	0.65 (0.52–0.81)	<0.001	0.74 (0.60–0.91)	0.005	2.85
sFlt-1/PlGF ratio (per 10-unit increase)	1.48 (1.25–1.75)	<0.001	1.35 (1.15–1.58)	<0.001	3.12

* Multivariable model was adjusted for gestational age at assessment in the training cohort (*n* = 210). AOR: adjusted odds ratio; VIF: variance inflation factor.

### 3.3 Construction and validation of the multimodal nomogram

Based on the identified independent predictors, a visual multimodal Nomogram was constructed to facilitate individualized risk assessment in clinical settings (Figure 4A). The model's calibration was rigorously evaluated. Both the training and internal validation cohorts demonstrated excellent agreement between the nomogram-predicted probabilities and the actual observed incidence of L-FGR (Figures 4B,C). The Brier scores were notably low (0.065 and 0.082, respectively). Furthermore, the Hosmer-Lemeshow goodness-of-fit test yielded a non-significant result (*χ*^2^ = 6.42, *P* = 0.601), visually confirmed by the alignment of expected and observed cases across risk deciles (Figure 4D), indicating the absence of substantial departure from perfect fit.

### 3.4 Diagnostic efficacy and subgroup robustness

The diagnostic performance of the combined model was benchmarked against single-modality approaches (Table 3). In the training cohort (*n* = 210), the combined multimodal model exhibited excellent discriminative power with an AUC of 0.948 (95% CI: 0.918–0.978), significantly exceeding that of the ultrasound-only model (AUC = 0.785, 95% CI: 0.721–0.849; DeLong *P* < 0.001) and serum-only model (AUC = 0.865, 95% CI: 0.812–0.918; DeLong *P* = 0.006) (Figure 5A). When evaluated in the internal validation cohort (*n* = 90), the combined model preserved robust discrimination with an AUC of 0.910 (95% CI: 0.845–0.965), with a sensitivity of 86.7% and specificity of 83.3%, which remained significantly superior to the ultrasound-only (AUC = 0.752, 95% CI: 0.648–0.856) and serum-only models (AUC = 0.835, 95% CI: 0.745–0.925) (Table 3, Figure 5B). Precision-Recall analysis further corroborated the superiority of the combined model (Average Precision [AP] = 0.932 in training and 0.875 in validation) (Figure 5C). Subgroup analysis confirmed that the model's predictive capability remained robust across early and late diagnostic gestational ages (Figure 5D).

**Table 3:** Diagnostic performance of prediction models in the training and internal validation cohorts.

Cohort/Model	AUC (95% CI)	Sensitivity (%)	Specificity (%)	PPV (%)	NPV (%)	Accuracy (%)
Training Cohort (*n* = 210)						
Ultrasound-only Model	0.785 (0.721–0.849)	67.1	75.7	58.0	82.2	72.9
Serum Biomarkers Model	0.865 (0.812–0.918)	80.0	79.3	64.4	89.2	79.5
Combined Multimodal Model	0.948 (0.918–0.978)	90.0	87.9	78.3	94.7	88.6
Validation Cohort (*n* = 90)						
Ultrasound-only Model	0.752 (0.648–0.856)	63.3	73.3	54.3	80.0	70.0
Serum Biomarkers Model	0.835 (0.745–0.925)	76.7	78.3	63.9	87.0	77.8
Combined Multimodal Model	0.910 (0.845–0.965)	86.7	83.3	72.2	92.6	84.4

**Figure 4:**
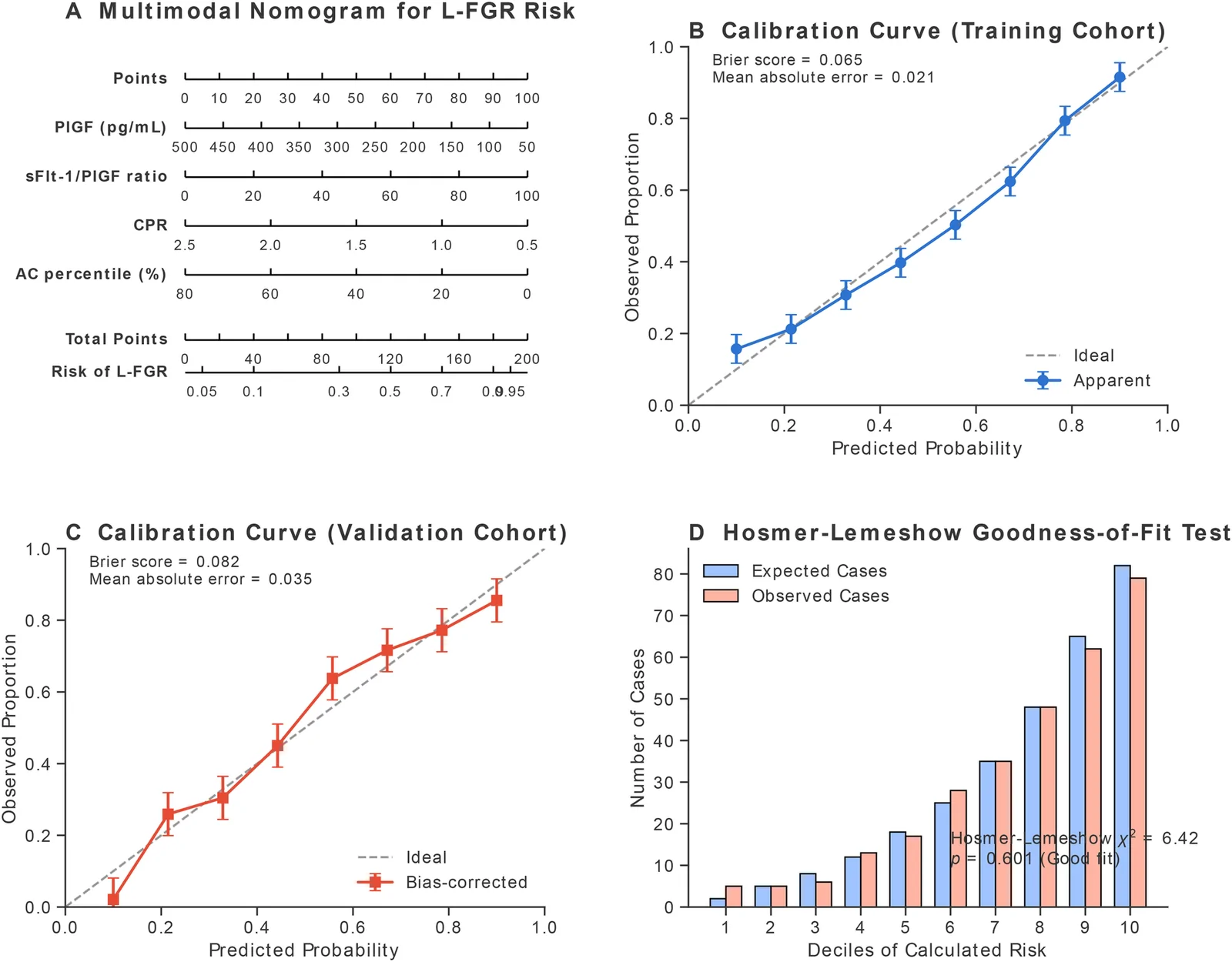
Construction and validation of the multimodal nomogram. **(A)** The developed multimodal nomogram incorporating independent ultrasound and serum variables for quantitative risk assessment and diagnostic evaluation of L-FGR. **(B)** Calibration curve for the prediction model in the training cohort. **(C)** Calibration curve in the internal validation cohort (using 1,000 bootstrap resamples). The dashed diagonal line represents the ideal prediction, and the solid line represents the model's apparent performance. **(D)** Hosmer-Lemeshow goodness-of-fit test visualized across risk deciles, showing the agreement between expected and observed L-FGR cases (*P* = 0.601).

### 3.5 Clinical utility and reclassification benefit

To translate predictive accuracy into clinical decision-making value, DCA was employed. As illustrated in Figure 6A, the combined model provided a superior net benefit across almost the entire range of threshold probabilities compared to the “treat-all,” “treat-none,” and single-modality strategies. The CIC further quantified this benefit, demonstrating a high degree of overlap between the number of patients deemed high-risk and the true positive cases, effectively minimizing unnecessary medical interventions (Figure 6B). Additionally, the integration of serum biomarkers into the traditional ultrasound model resulted in a significant continuous NRI of 0.452 and an Integrated Discrimination Improvement (IDI) of 0.128, confirming a substantial improvement in risk stratification (Figure 6D).

**Figure 5:**
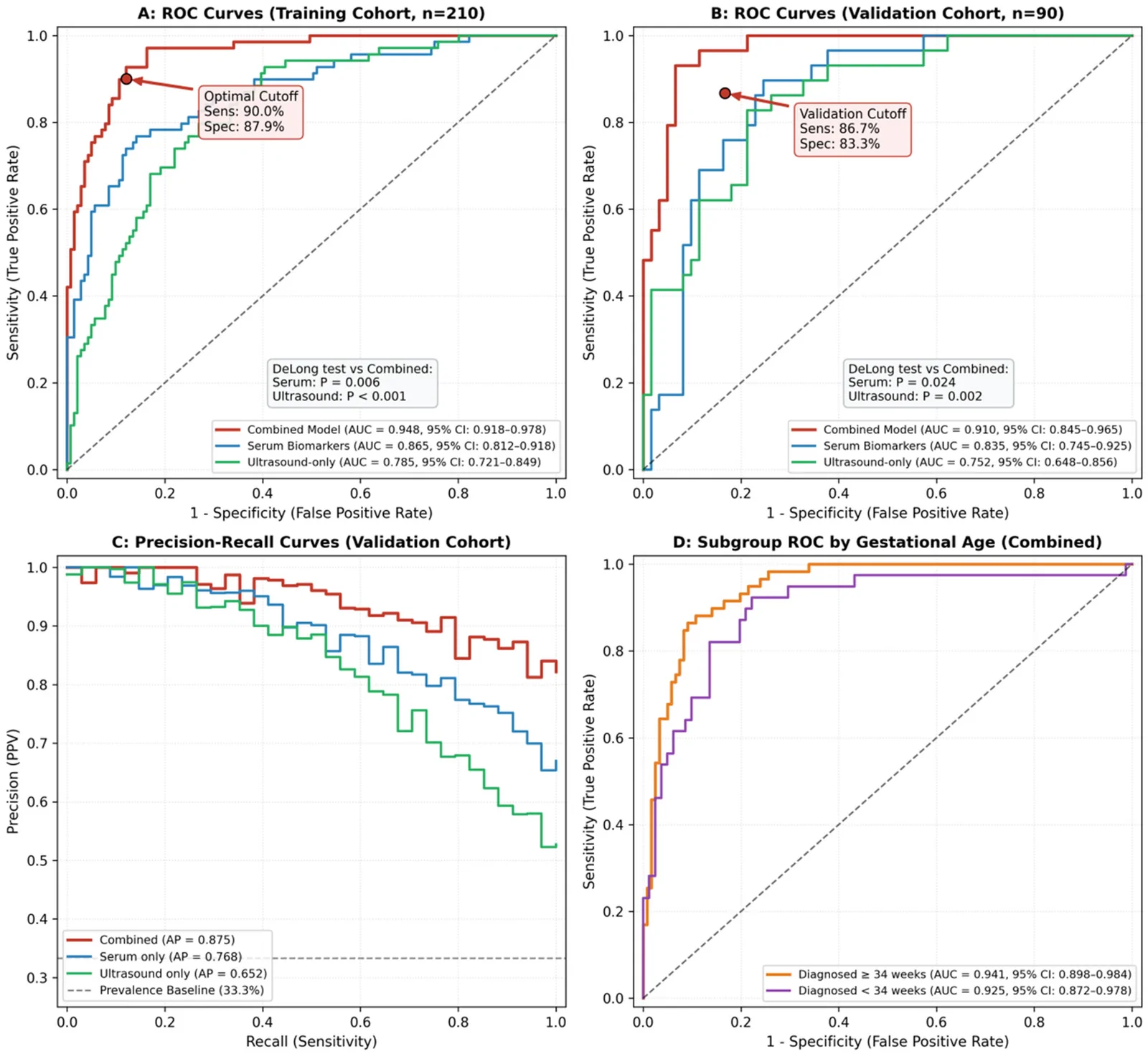
Diagnostic performance and subgroup analysis. **(A)** Receiver Operating Characteristic (ROC) curve of the combined multimodal diagnostic-support model. **(B)** Comparison of ROC curves among the ultrasound-only model, serum-biomarker-only model, and the combined model. *P* values were calculated using the DeLong test. **(C)** Precision-Recall (PR) curves comparing the models’ performance in handling imbalanced positive cases. **(D)** Subgroup ROC analysis stratified by gestational age at diagnosis.

### 3.6 Prediction of adverse perinatal outcomes

A critical objective of this model is its prognostic value for adverse perinatal endpoints. Applying the optimal probability cutoff established in the training cohort to the entire study population (*N* = 300), patients were stratified into high-risk (*n* = 95) and low-risk (*n* = 205) groups. Kaplan–Meier survival analysis revealed a significantly accelerated time-to-delivery in the high-risk cohort (Log-rank test, *P* < 0.001), indicating a higher probability of requiring premature obstetrical intervention (Figure 7A). As detailed in Table 4, the incidence rates of NICU admission, emergency cesarean section, and neonatal hypoglycemia were all drastically elevated in the high-risk group (Figure 7B).

**Figure 6:**
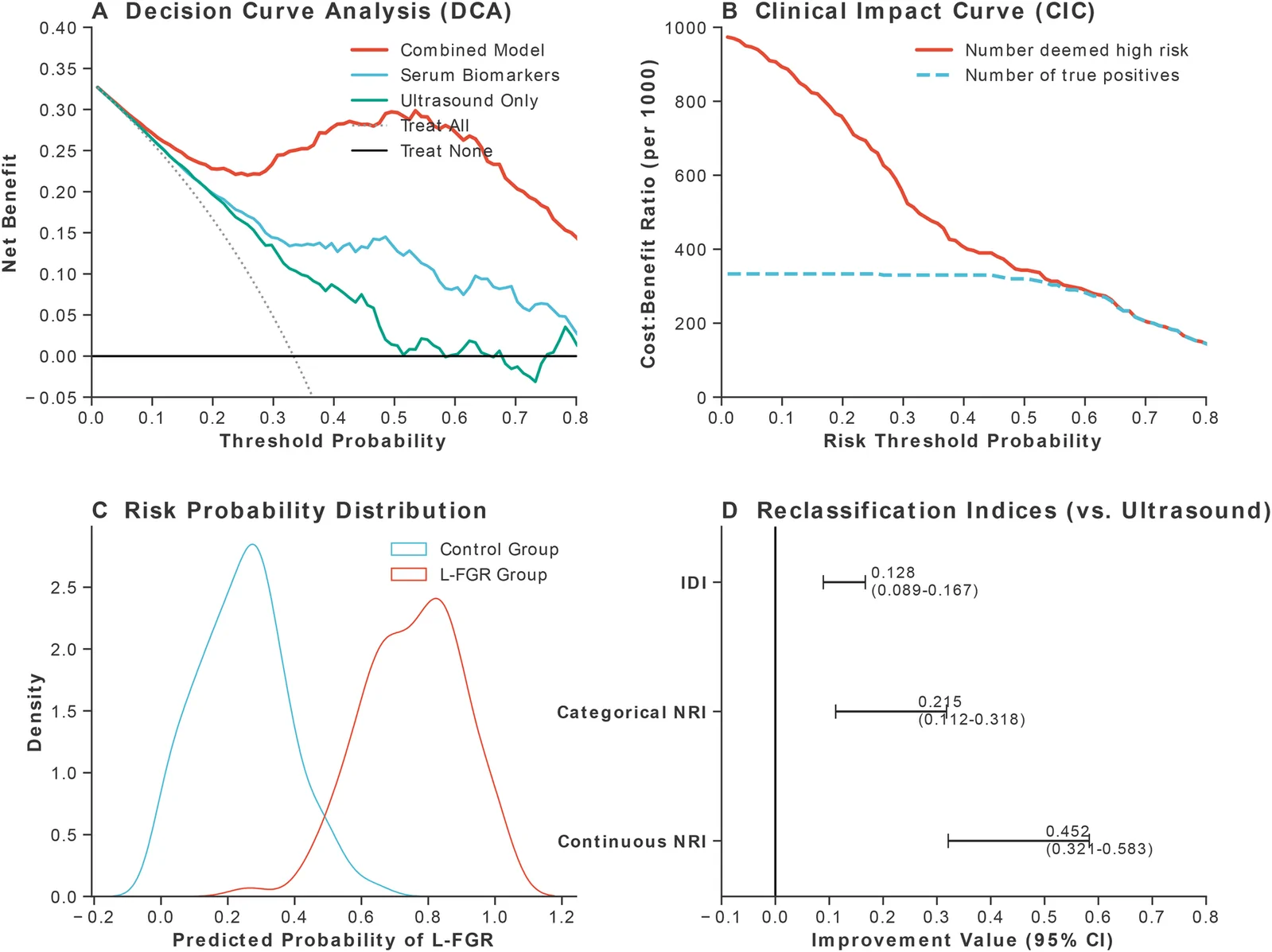
Clinical utility and reclassification evaluation. **(A)** Decision Curve Analysis (DCA) demonstrating the net benefit of different prediction models across various threshold probabilities. **(B)** Clinical Impact Curve (CIC) of the combined model, showing the estimated number of patients deemed high-risk versus the true positive cases per 1,000 pregnancies. **(C)** Density plot illustrating the risk probability distribution between the L-FGR and control groups. **(D)** Net Reclassification Index (NRI) and Integrated Discrimination Improvement (IDI) confirming the added value of integrating serum biomarkers into the traditional ultrasound model.

**Table 4:** Incidence of adverse perinatal outcomes stratified by model risk groups in the total study cohort (*N* = 300).

Outcomes	Low-Risk Group (*n* = 205)	High-Risk Group (*n* = 95)	*P*-value
NICU Admission, *n* (%)	26 (12.7%)	43 (45.3%)	< 0.001
Emergency C-section, *n* (%)	41 (20.0%)	55 (57.9%)	< 0.001
Neonatal Hypoglycemia, *n* (%)	8 (3.9%)	18 (18.9%)	< 0.001

Risk stratification was evaluated across the entire enrolled cohort (*N* = 300, comprising 205 low-risk and 95 high-risk pregnancies) using the optimal cutoff probability threshold determined by the maximum Youden index derived from the training cohort (*n* = 210). High-risk fetuses exhibited significantly accelerated delivery and elevated adverse outcomes. Values are presented as numbers (percentages). *P*-values were calculated using Pearson's Chi-square test or Fisher's exact test.

### 3.7 Benchmarking against machine learning algorithms

To benchmark against machine learning standards, the discriminative and clinical performance of the multivariable Logistic Regression (LR) model was compared against state-of-the-art machine learning algorithms, including eXtreme Gradient Boosting (XGBoost), Random Forest (RF), and Support Vector Machine (SVM). To ensure unbiased evaluation, all hyperparameter tuning was conducted via 5-fold cross-validated grid search within the training set, and final benchmarking was performed strictly on the independent validation cohort (*n* = 90). The interpretable LR nomogram demonstrated an AUC of 0.910 (95% CI: 0.845–0.965), which was highly comparable to ensemble architectures (XGBoost: AUC = 0.924, 95% CI: 0.865–0.983; Random Forest: AUC = 0.898, 95% CI: 0.830–0.966) and notably outperformed SVM (AUC = 0.831, 95% CI: 0.742–0.920) (Figure 8A). Consistent with the ROC analysis, Precision-Recall benchmarking confirmed robust efficacy across positive classes (Average Precision: XGBoost = 0.892, LR = 0.875, RF = 0.841, SVM = 0.756) (Figure 8B). Furthermore, Decision Curve Analysis indicated that the transparent LR nomogram achieved clinical net benefit equivalent to complex black-box algorithms across a wide range of threshold probabilities (Figure 8C).

**Figure 7:**
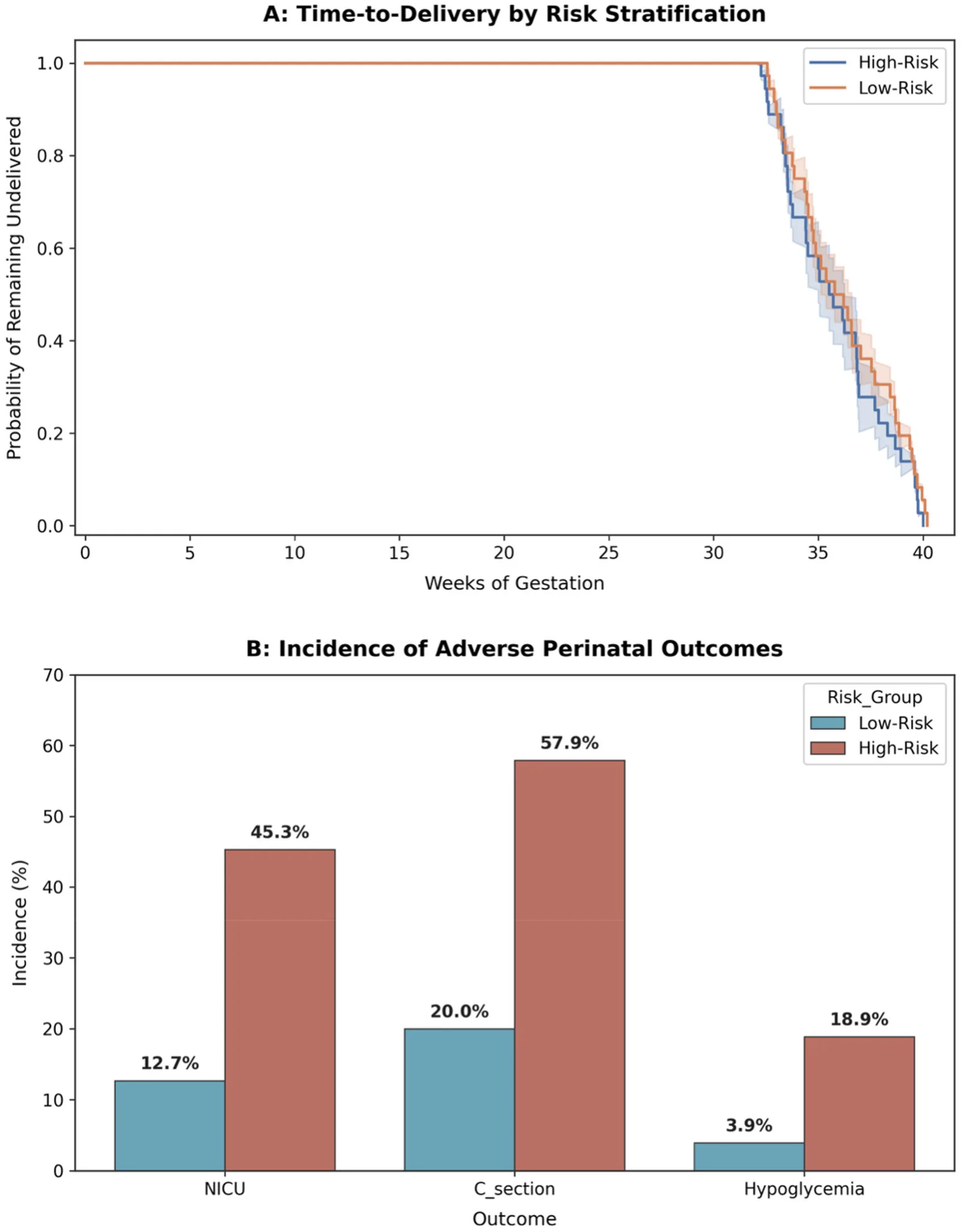
Prognostic value for adverse perinatal outcomes. **(A)** Kaplan–Meier curves for the probability of remaining undelivered, stratified by the model-derived low-risk and high-risk groups. **(B)** Incidence rates of severe adverse perinatal outcomes (NICU admission, emergency C-section, and neonatal hypoglycemia) between risk groups.

**Figure 8:**
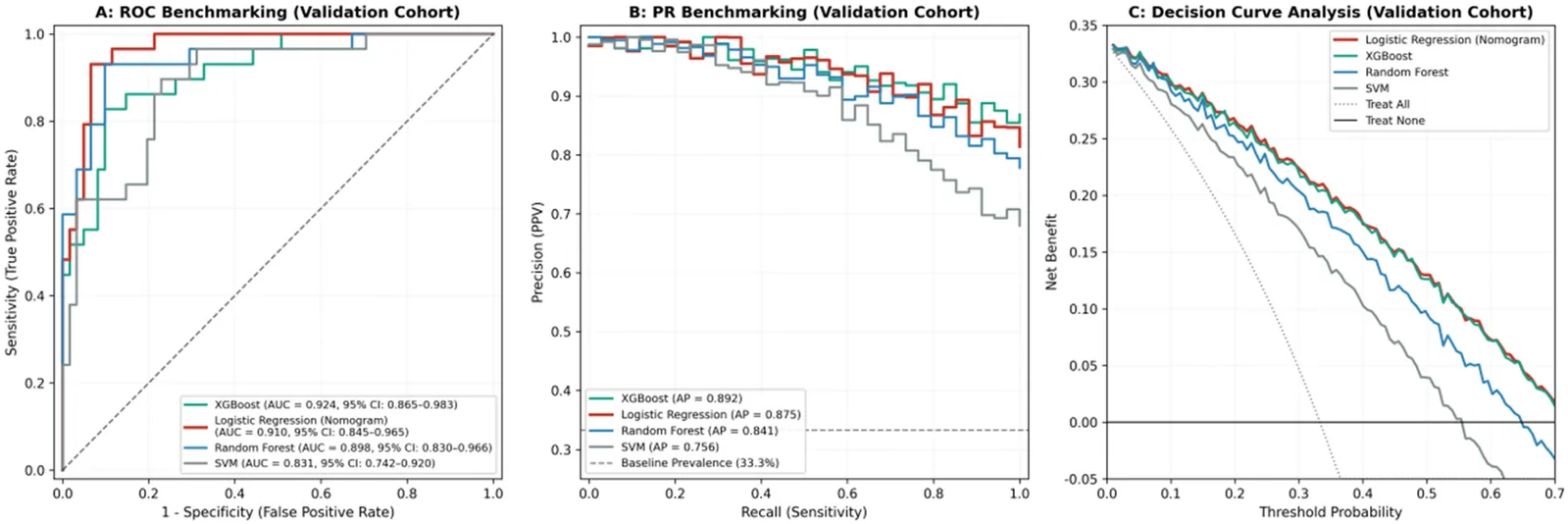
Performance benchmarking against advanced machine learning algorithms. **(A)** ROC curves comparing the standard Logistic Regression model against state-of-the-art machine learning algorithms (XGBoost, Random Forest, and SVM). **(B)** Precision-Recall curves comparison. **(C)** Decision Curve Analysis confirming that the highly interpretable Logistic Regression model provides a clinical net benefit equivalent to complex, black-box machine learning algorithms.

## 4 Discussion

In this study, we successfully developed and internally validated a highly robust, multimodal diagnostic-support nomogram for the risk stratification of late-onset fetal growth restriction (L-FGR) and its subsequent adverse perinatal outcomes. By synergistically integrating maternal serum biomarkers (PlGF and the sFlt-1/PlGF ratio) with traditional fetal ultrasound parameters (AC percentile and CPR), our combined model achieved an exceptional discriminative capability (AUC = 0.910), significantly outperforming conventional single-modality approaches. Furthermore, the model demonstrated excellent calibration and substantial clinical net benefit. Importantly, the nomogram effectively stratified patients at high risk for composite adverse perinatal endpoints, thereby providing a highly interpretable and pragmatic tool to optimize obstetric management.

The clinical diagnosis and management of L-FGR remain notoriously challenging (1, 2). Unlike early-onset FGR, which is typically characterized by severe placental insufficiency and easily detectable abnormal umbilical artery (UA) Doppler velocimetry, L-FGR often presents with subtle morphometric deviations and normal UA blood flow until the terminal stages (3, 6). Consequently, relying solely on estimated fetal weight or abdominal circumference leads to suboptimal detection rates. In our cohort, the inclusion of the cerebroplacental ratio (CPR) significantly improved the detection of fetal hypoxemia, reflecting the “brain-sparing” effect (8, 10). However, hemodynamic redistribution is a downstream phenotypic consequence of placental dysfunction. To capture the upstream pathophysiological etiology, we incorporated the sFlt-1/PlGF ratio (11, 13). The abnormal elevation of the sFlt-1/PlGF ratio and the depletion of PlGF reflect placental syncytiotrophoblast stress, subclinical malperfusion, and an anti-angiogenic state (14, 15). By combining the biochemical indicators of placental dysfunction with the sonographic signs of fetal adaptation, our multimodal model captures the complete pathophysiological spectrum of L-FGR, which explains its superior diagnostic performance and the substantial improvement in the Net Reclassification Index (NRI) (21).

Beyond diagnostic accuracy, the ultimate value of any clinical prediction model hinges on its capacity to facilitate informed decision-making (19, 20). In contemporary obstetric practice, clinicians constantly face the dilemma of balancing the risk of unexplained intrauterine fetal demise against the morbidities associated with iatrogenic prematurity (22). Our Decision Curve Analysis (DCA) and Clinical Impact Curve (CIC) compellingly demonstrated that utilizing this nomogram yields a higher net benefit across a wide range of threshold probabilities compared to the “treat-all” or “treat-none” strategies. More crucially, the model exhibits profound prognostic value. Kaplan–Meier survival analysis and the complication matrix revealed that fetuses classified into the high-risk group by our nomogram faced a drastically accelerated time-to-delivery and a markedly higher incidence of severe composite adverse outcomes (4, 23). Notably, secondary clinical endpoints such as emergency cesarean delivery and time-to-delivery are inherently management-dependent outcomes. Because clinicians were not blinded to ultrasound Doppler findings and diagnostic classifications, fetal surveillance frequency and expedited delivery decisions may have been directly influenced by the established L-FGR diagnosis rather than spontaneous pathological deterioration alone. This potential ascertainment bias warrants confirmation through prospective double-blind management trials. This suggests that our nomogram is not merely a diagnostic calculator, but a vital prognostic triage tool that can guide the frequency of fetal surveillance and optimize the timing of delivery.

A notable methodological strength of this study is the benchmarking of our standard logistic regression (LR) model against state-of-the-art machine learning (ML) algorithms (24, 25). While advanced ML techniques, such as eXtreme Gradient Boosting (XGBoost) and Random Forest, possess an intrinsic advantage in capturing complex, non-linear interactions within high-dimensional data, their “black-box” nature inherently limits clinical interpretability and physician trust (26, 27). Our comparative analysis revealed that the predictive efficacy and clinical net benefit of the LR model were highly comparable to those of XGBoost. Therefore, considering the equivalent performance and the superior transparency offered by the visual nomogram, the LR-based architecture remains the most pragmatic, reliable, and user-friendly choice for daily obstetric triage (28).

A critical methodological consideration in this study pertains to potential incorporation bias. Under the ISUOG Delphi consensus criteria, fetal biometry (such as AC percentile) and hemodynamics (such as CPR) represent constitutive elements of the diagnostic reference standard for L-FGR. Evaluating these parameters within a multivariable model to classify L-FGR inherently introduces circularity, which inflates standalone discrimination. In this context, our nomogram should not be conceptualized as an early *a priori* screening tool, but rather as a multimodal diagnostic-support and quantitative risk-stratification instrument. Crucially, our findings demonstrate that incorporating maternal angiogenic biomarkers (the sFlt-1/PlGF ratio and PlGF) provides substantial incremental value over sonographic biometry and Doppler alone, as formally substantiated by significant Net Reclassification Improvement (NRI = 0.452) and Integrated Discrimination Improvement (IDI = 0.128). This multimodal synthesis effectively addresses the clinical ‘gray zone’ of late gestation, aiding in both concurrent diagnostic triage and subsequent prognostic risk stratification for adverse perinatal endpoints (29, 30).

Despite the robust methodology and promising results, several limitations of this study must be acknowledged. First, the retrospective, single-center design may introduce inherent selection biases and limit the generalizability of the findings to broader populations. Second, the relatively modest sample size (*N* = 300) might have restricted the statistical power to detect differences in extremely rare, albeit severe, neonatal complications. Third, our study relied on single-timepoint maternal serum biomarker measurements obtained concurrently with the late ultrasound exam. Given that angiogenic markers undergo physiological dynamics across gestation and that our case cohort was diagnosed slightly earlier than controls, gestational-age adjustment was implemented in regression analyses. Nonetheless, unstandardized absolute values rather than gestation-specific Multiples of the Median (MoM) were utilized, which may leave residual confounding from gestational age. Future prospective studies tracking longitudinal biomarker trajectories will be valuable to validate these findings. Furthermore, large-scale, prospective, multicenter cohorts are mandatory to externally validate the generalizability and clinical safety of this nomogram before routine bedside adoption.

## 5 Conclusion

In conclusion, we developed a highly accurate and clinically actionable multimodal nomogram that integrates maternal serum angiogenic biomarkers with fetal ultrasound hemodynamics for the risk stratification and clinical evaluation of L-FGR. This user-friendly tool significantly enhances risk stratification for adverse perinatal outcomes, empowering obstetricians to make personalized, evidence-based decisions regarding fetal surveillance and the optimal timing of delivery.

## Data Availability

The raw data supporting the conclusions of this article will be made available by the authors, without undue reservation.
